# Not all that thickens is hypertrophic cardiomyopathy: a case report of unusual Takotsubo cardiomyopathy

**DOI:** 10.1093/ehjcr/ytaf427

**Published:** 2025-08-29

**Authors:** Ibrahim Antoun, Sanjay S Bhandari

**Affiliations:** Department of Cardiology, University Hospitals of Leicester NHS Trust, Groby Road, Glenfield Hospital, Leicester LE3 0QP, UK; Department of Cardiovascular Sciences, University of Leicester, Groby Road, Glenfield Hospital, Leicester LE3 9QP, UK; Department of Cardiology, University Hospitals of Leicester NHS Trust, Groby Road, Glenfield Hospital, Leicester LE3 0QP, UK

**Keywords:** Takotsubo cardiomyopathy, Hypertrophic cardiomyopathy, Acute coronary syndrome, Echocardiography, Cardiac magnetic resonance, Case Report

## Abstract

**Background:**

Takotsubo cardiomyopathy (TTC) is an acute, reversible syndrome of left ventricular dysfunction typically triggered by stress and characterized by regional wall motion abnormalities often mimicking acute coronary syndrome. Although classically associated with apical ballooning, TTC can present with atypical patterns and may mimic structural cardiomyopathies, particularly apical hypertrophic cardiomyopathy (HCM). Transient myocardial oedema in TTC can result in reversible wall thickening and high signal intensity on cardiac magnetic resonance imaging, producing a pseudohypertrophic appearance that resembles HCM—a diagnostic pitfall with significant clinical implications.

**Case summary:**

We report a 74-year-old woman presenting with dyspnoea, elevated cardiac biomarkers, and echocardiographic apical ballooning. Coronary angiography revealed unobstructed coronary arteries, prompting consideration of TTC. Cardiac magnetic resonance performed 3 days later demonstrated global myocardial oedema, with disproportionate apical signal and cavity obliteration during systole mimicking HCM but no late gadolinium enhancement or infarction. The initial apical ballooning had resolved. The diagnosis of TTC with transient pseudohypertrophy due to myocardial oedema was made. The patient was commenced on guideline-directed heart failure therapy.

**Discussion:**

This case underscores the diagnostic complexity at the intersection of TTC, myocardial infarction with non-obstructive coronary arteries, and HCM mimics. Cardiac magnetic resonance was pivotal in revealing myocardial oedema and excluding infarction or fibrosis. Reversible wall thickening in TTC may resemble apical HCM, a phenomenon termed pseudohypertrophy. Takotsubo cardiomyopathy should remain a key differential despite angiographic findings. Early multimodal imaging is crucial for avoiding misdiagnosis and guiding appropriate management.

Learning points

- Takotsubo cardiomyopathy can mimic apical hypertrophic cardiomyopathy on cardiac MRI due to transient myocardial oedema and wall thickening, a phenomenon known as pseudohypertrophy.
- Cardiac magnetic resonance imaging, particularly T1 mapping and late gadolinium enhancement, is essential for differentiating Takotsubo cardiomyopathy from structural cardiomyopathies and infarction.

## Introduction

Cardiovascular diseases are increasing in prevalence and its management is a challenge especially in the developing world.^1,2^ Takotsubo cardiomyopathy (TTC), also referred to as stress-induced cardiomyopathy or ‘broken heart syndrome,’ is a transient cardiac syndrome characterized by acute but reversible left ventricular (LV) systolic dysfunction in the absence of significant coronary artery obstruction. First described in Japan in the 1990s, it has since been increasingly recognized worldwide, particularly in postmenopausal women presenting with symptoms and biomarkers that mimic acute coronary syndrome (ACS).^3,4^ The syndrome is typically triggered by physical or emotional stress and is associated with characteristic regional wall motion abnormalities, most commonly involving apical ballooning. However, atypical variants involving mid-ventricular or basal segments have also been described. The diagnosis of TTC is complex and frequently relies on a combination of clinical presentation, electrocardiogram (ECG) findings, cardiac biomarker elevation, coronary angiography, and advanced cardiac imaging such as cardiac magnetic resonance (CMR).

Although the underlying mechanisms of TTC are not fully understood, catecholamine-induced myocardial stunning is thought to play a central role. More recently, recognizing diffuse myocardial oedema during the acute phase—detectable via CMR—has expanded our understanding of TTC’s pathophysiology and its potential to mimic other cardiac disorders, particularly apical hypertrophic cardiomyopathy (HCM).^5^ Importantly, TTC may coexist with or be masked by obstructive coronary artery disease (CAD),^4^ further complicating diagnosis, especially in elderly patients with multiple comorbidities. Moreover, the overlap with the clinical spectrum of myocardial infarction with non-obstructive coronary arteries (MINOCA) underscores the need for a multimodal diagnostic approach. CMR imaging plays a central role in elucidating the underlying myocardial tissue characteristics, enabling assessment of oedema, fibrosis, and infarction patterns.^6^

The diagnostic spectrum of TTC increasingly overlaps with other conditions that present with acute chest symptoms, non-obstructive coronaries, and elevated cardiac biomarkers. One particularly challenging mimic is apical hypertrophic cardiomyopathy (HCM), especially when transient myocardial oedema in TTC leads to reversible increases in wall thickness and altered myocardial signal on CMR imaging—a phenomenon sometimes termed pseudohypertrophy. This pseudohypertrophic appearance can closely resemble the structural abnormalities of true HCM, potentially leading to misdiagnosis and inappropriate long-term management strategies. In such complex scenarios, advanced imaging techniques—particularly CMR—play a critical role in tissue characterization and in distinguishing transient myocardial processes from structural cardiomyopathies or infarction. The ability of CMR to assess oedema, late gadolinium enhancement (LGE), and wall motion abnormalities over time is especially valuable in patients presenting with myocardial infarction with non-obstructive coronary arteries (MINOCA), a syndrome under which TTC is now recognized. In this context, our report aims to highlight the diagnostic challenges in distinguishing TTC from apical HCM using multimodal imaging and to emphasize the importance of recognizing pseudohypertrophy in the acute phase of TTC. This has critical implications for prognosis, therapeutic decisions, and follow-up strategies.

## Summary figure

**Figure:**
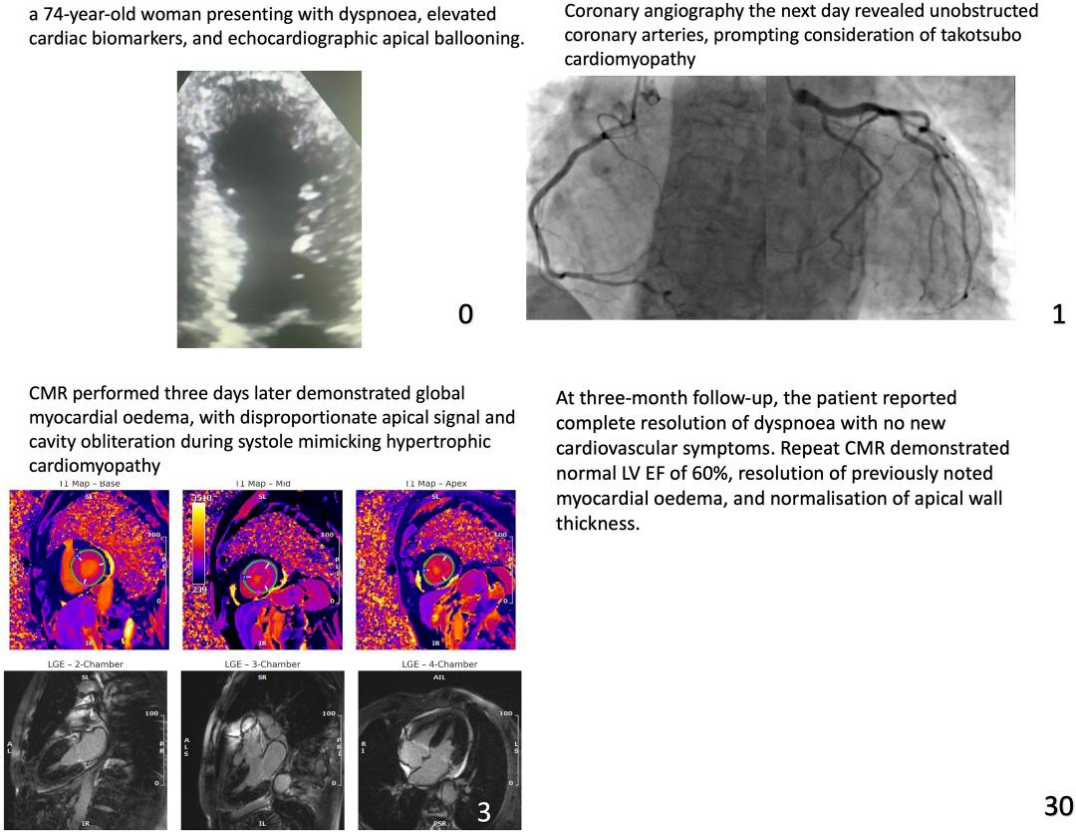


## Case presentation

**Figure 1:**
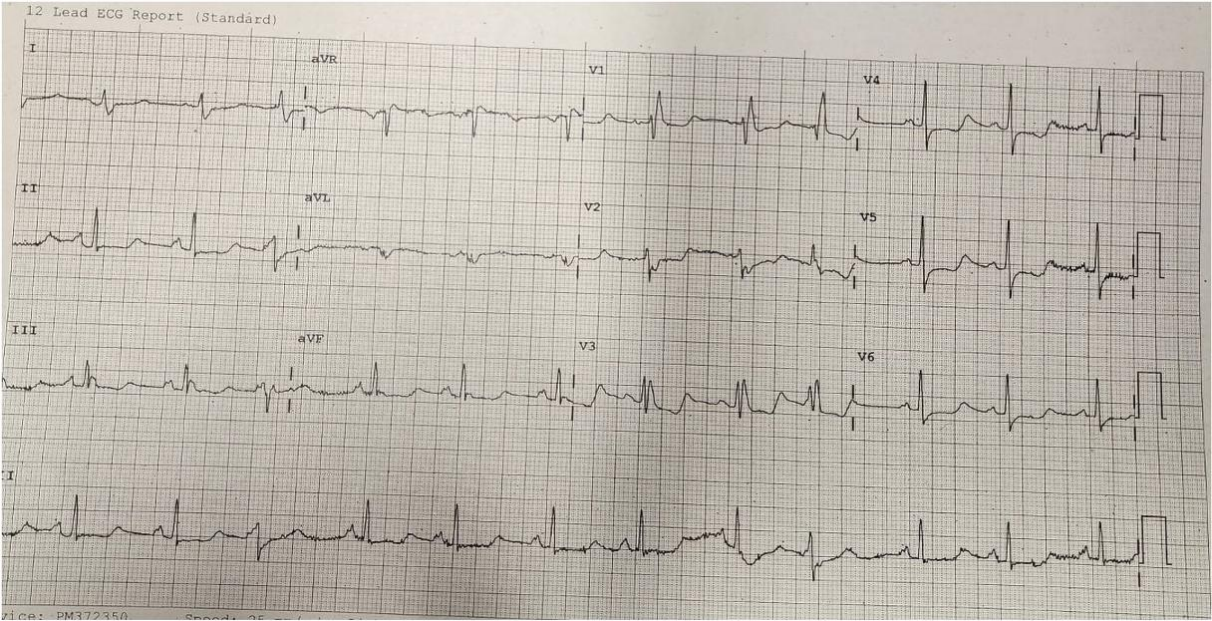
Twelve-lead electrocardiogram on admission demonstrating right bundle branch block morphology without dynamic ST changes.

**Figure 2:**
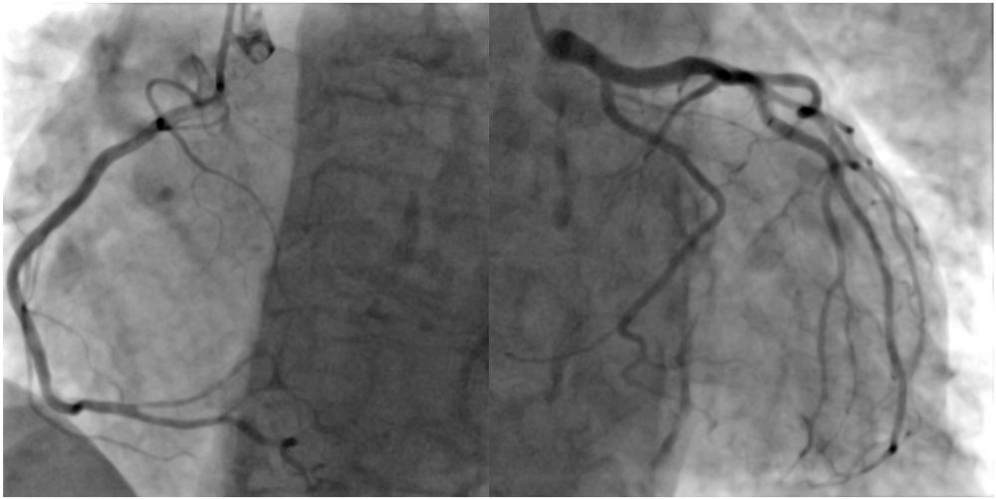
Invasive coronary angiogram demonstrating no obstructive coronary artery disease.

**Figure 3:**
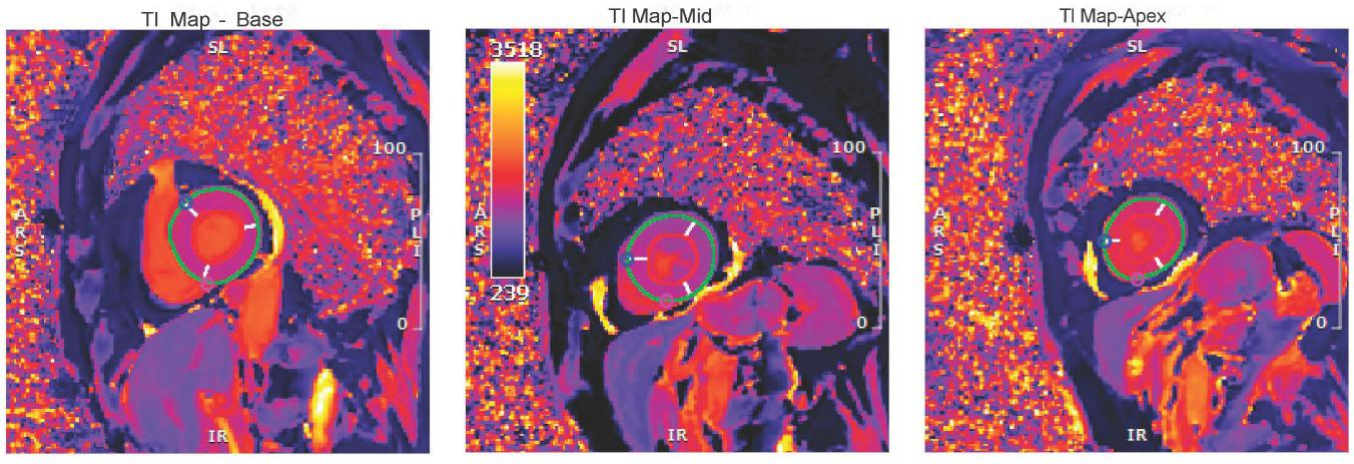
T1 mapping in short-axis views at base, mid, and apex. Parametric maps demonstrate globally elevated myocardial T1 values consistent with diffuse myocardial oedema. Notably, the apical segment (right panel) shows markedly increased T1 signal intensity compared to the base and mid-ventricle, creating a pseudohypertrophic appearance mimicking apical hypertrophic cardiomyopathy (HCM). In the absence of late gadolinium enhancement, this finding supports a diagnosis of Takotsubo cardiomyopathy (TTC) with apical predominance.

**Figure 4:**
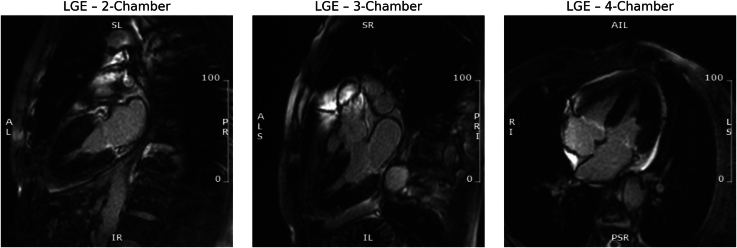
Late gadolinium enhancement (LGE) imaging in long-axis views. Two-chamber, three-chamber, and four-chamber CMR views reveal no evidence of myocardial enhancement, confirming the absence of myocardial fibrosis or infarction. These findings support the diagnosis of Takotsubo cardiomyopathy rather than apical hypertrophic cardiomyopathy or ischaemic injury. The absence of LGE, despite elevated troponin and severe left ventricular dysfunction, aligns with a reversible myocardial injury pattern.

Our patient is a 74-year-old female who presented to the emergency department due to shortness of breath for the past 2 days (Video 1). She has a background history of a cerebrovascular event and hypertension. Her cardiovascular examination yielded a clear chest and normal heart sounds. Her vital signs were satisfactory, and the ECG showed sinus rhythm with right bundle branch block. The patient did not recall any recent emotional or physical stressor before symptom onset. There were no notable ST-segment changes (*Figure 1*). Her blood results yielded a high-sensitivity troponin I level of 659 ng/L (reference < 10 ng/L), which increased to 2160 ng/L four hours later. Her N-terminal pro B-type natriuretic peptide (NT-Pro BNP) was 3905 ng/L (reference: 20–200 ng/L). Her white cell count was 15.6 × 10^9^/L, and her C-reactive protein was <10 mg/L. Her chest X-ray was satisfactory, and her transthoracic echocardiogram (TTE) demonstrated an impaired left ventricular (LV) systolic function with ejection fraction (EF) of 35% by visual estimation. There was apical ballooning with akinesia. The patient underwent an invasive coronary angiogram (Video 1, *Figure 2*), which showed unobstructed coronary arteries, raising the possibility of TCC. The patient underwent CMR according to MINOCA protocol 3 days after the TTE. This showed the resolution of the apical ballooning but increased T1 signals, indicating global myocardial oedema with a more intense signal in the apical segments (*Figure 3*). There was also apical cavity obliteration during systole mimicking HCM (Video 2). LV EF was 44%, and there was no evidence of late gadolinium (LGE) enhancement (*Figure 4*). The patient went into atrial fibrillation, which was rate-controlled and anticoagulated appropriately. Serial high-sensitivity troponin I levels peaked at 2160 ng/L and gradually declined to 425 ng/L before discharge. By discharge, the high-sensitivity troponin I level had decreased to 425 ng/L, and the NT-proBNP level had fallen to 1100 ng/L.

The patient was initiated on guideline-directed medical therapy for heart failure with reduced ejection fraction, including bisoprolol 2.5 mg once daily, ramipril 2.5 mg once daily (up-titrated as tolerated), and furosemide 20 mg orally as needed for volume control. Anticoagulation with apixaban 5 mg twice daily was also commenced. At three-month follow-up, the patient reported complete resolution of dyspnoea with no new cardiovascular symptoms. Repeat CMR demonstrated normal LV EF of 60%, resolution of previously noted myocardial oedema, and normalization of apical wall thickness. There was no LGE or residual wall motion abnormality.

## Discussion

This case illustrates the complex interplay between TCC and HCM phenocopies in an elderly patient with elevated biomarkers and LV dysfunction. The initial clinical and echocardiographic presentation suggested TTC—with apical ballooning and severely impaired systolic function. Subsequently, CMR provided crucial diagnostic clarification, revealing diffuse myocardial oedema, resolution of apical wall motion abnormalities, and absence of infarction, consistent with TTC.

Notably, the CMR findings demonstrated global myocardial oedema, with disproportionate signal intensity in the apical segments that transiently mimicked the imaging features of apical HCM. This raises a critical diagnostic consideration: TTC can present with reversible increases in myocardial wall thickness due to extensive interstitial oedema, especially in the acute phase. This phenomenon has been termed *pseudohypertrophy* and described in several CMR-based studies as a reversible mimic of hypertrophic phenotypes.^7,8^ In our patient, the intense apical signal and increased wall thickness on CMR, in the absence of late LGH, raised concern for an apical HCM-like appearance, but the absence of myofibre disarray or fibrosis—as would be seen in true HCM—favoured a TTC diagnosis.

CMR is particularly valuable in such cases due to its ability to characterize myocardial tissue and differentiate between oedema, fibrosis, and infarction. T2-weighted sequences and T2 mapping are sensitive to myocardial oedema, a hallmark of the acute phase of TTC, while LGE imaging assesses myocardial scarring. In HCM, LGE is typically observed in areas of hypertrophy, often involving the interventricular septum or apex, with a patchy or diffuse distribution.^9^ In contrast, TTC typically lacks LGE or demonstrates only minimal non-ischaemic enhancement, and wall thickening seen on cine imaging resolves over time. Our patient’s apical thickening without LGE and normalization of wall motion supported TTC with transient apical oedema rather than primary myocyte hypertrophy. The absence of apical ballooning on CMR, despite its presence on initial echocardiography. This reflects the dynamic nature of wall motion abnormalities in TTC, which typically evolve rapidly and may begin to resolve within 48–72 h after symptom onset. Several studies have reported that regional dysfunction may be transient and partially or fully resolved by the time CMR is performed, particularly if imaging is delayed beyond the acute phase.^6,10^ In our patient, CMR was performed on Day 3, at which point apical contractility had normalized. This temporal dissociation underscores the importance of early imaging and reinforces the utility of tissue characterization—particularly myocardial oedema—as a more persistent and diagnostically informative marker in subacute presentations. Although true HCM was not present, the transient increase in apical wall thickness and systolic cavity obliteration seen on CMR mimicked the imaging phenotype of apical HCM. This phenomenon, previously described as pseudohypertrophy, is attributable to interstitial myocardial oedema in the acute phase of TTC.^7^ We acknowledge that cavity obliteration alone is not diagnostic of HCM, and our imaging findings did not demonstrate the hallmark features of HCM, such as myofibre disarray, asymmetric hypertrophy, or LGE. Rather, this case underscores how TTC may transiently simulate features of structural cardiomyopathy, highlighting the importance of repeat imaging to distinguish phenocopies from true cardiomyopathies.

This diagnostic overlap is not merely academic but has important prognostic and therapeutic implications. A misdiagnosis of apical HCM could lead to unnecessary long-term therapy and follow-up for structural cardiomyopathy. At the same time, TTC generally has a favourable prognosis with supportive treatment, although recurrence remains a concern in a minority of cases.^4^

Beyond imaging findings, this case also highlights the importance of structured diagnostic reasoning in patients with elevated troponin and unobstructed coronaries. In line with the European Society of Cardiology (ESC) recommendations for MINOCA evaluation,^11^ our diagnostic pathway incorporated early CMR to distinguish between ischaemic and non-ischaemic causes of myocardial injury. While not formally calculated at the time, the patient’s clinical features—including postmenopausal status, absence of ST-segment depression, and physical trigger—would have yielded an InterTAK Diagnostic Score > 50, strongly suggestive of TTC.^12^ However, in our patient, the absence of a clear emotional or physical stressor and the unusual CMR findings raised diagnostic uncertainty, justifying the decision to prioritize tissue characterization and delay definitive diagnosis until follow-up imaging. This stepwise, multimodal approach—though deviating from conventional algorithmic diagnosis—ultimately avoided misclassification and unnecessary treatment for structural cardiomyopathy. Such real-world nuances emphasize the importance of balancing structured diagnostic tools with clinical judgement and imaging timing. Our patient underwent CMR within the recommended timeframe, and using a dedicated MINOCA protocol was instrumental in avoiding diagnostic ambiguity. Follow-up imaging remains essential to confirm the resolution of wall motion abnormalities and oedema and rule out residual structural cardiomyopathy.

This case highlights the crucial role of CMR in distinguishing TCC from structural mimics, such as apical HCM, particularly when transient apical oedema leads to pseudohypertrophy. The overlap in imaging features necessitates a cautious, temporally guided diagnostic approach, particularly regarding reversibility, oedema patterns, and LGE distribution. In elderly patients with cardiovascular risk factors and coexisting CAD, TTC should remain a key differential diagnosis, and CMR can provide diagnostic resolution in challenging MINOCA-like presentations.

## Lead author biography


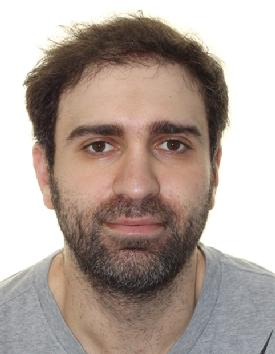


Graduated from University of Aleppo with MD degree in 2016. UK licensed physician and a member of the Royal College of Physicians in the UK in 2021. Completed his PhD in Electrophysiology at the University of Leicester in 2023. Currently works as a Cardiology trainee in East Midlands deanery and an honorary research fellow at the University of Leicester.

## Data Availability

Data regarding this case report is available on request from the corresponding author.
